# Protease mutations emerging on darunavir in protease inhibitor-naïve and experienced patients in the UK

**DOI:** 10.7448/IAS.17.4.19739

**Published:** 2014-11-02

**Authors:** Kate El Bouzidi, Ellen White, Jean L Mbisa, Andrew Phillips, Nicola Mackie, Anton Pozniak, David Dunn

**Affiliations:** 1 Department of Virology, University College London, London, UK; 2 MRC Clinical Trials Unit, Medical Research Council, London, UK; 3 Antiviral Unit, Public Health England, London, UK; 4 Infection & Population Health, University College London, London, UK; 5 Imperial College Healthcare NHS Trust, Jefferiss Wing, St Mary's Hospital, London, UK; 6 HIV and Sexual Health, Chelsea & Westminster Hospital NHS Trust, London, UK; 7 MRC Clinical Trials Unit, University College London, London, UK

## Abstract

**Introduction:**

Darunavir (DRV) is a preferred agent in treatment guidelines for ART-naïve and experienced patients [1]. It is considered to have a high genetic barrier to resistance and 11 resistance-associated mutations (RAMs) are recognized by IAS-USA [2]. These have largely been identified by analyses examining the correlation between baseline genotype and virological response [3]. However, there is little information on RAMs that are directly selected by DRV, outside of short-term clinical trials. We aimed to identify emerging mutations by comparing the genotypes of individuals before and after DRV exposure.

**Materials and Methods:**

The UK HIV Drug Resistance Database was used to identify patients aged over 16 who had received at least 30 days of a DRV-containing regimen. Patients were included if they had a “baseline” resistance test, prior to DRV exposure, and a “repeat” test, either on DRV or within 30 days of stopping this agent. To avoid attributing the effects of other PIs on emerging RAMs to DRV, patients were excluded if they had received another PI for greater than 90 days between the baseline genotype and the start of DRV. The baseline and repeat tests were compared to determine the nature of mutations stratified by PI history.

**Results:**

A total of 5623 patients had DRV, of whom 306 met the inclusion criteria. A total of 228 (74.5%) were male, median age at the start of DRV was 42 years (IQR 37–47), and half had subtype B infection. The mode of transmission was homosexual contact for 50%, heterosexual for 38%, and 3% were injection drug users. The median CD4 count at the start of DRV was 257 cells/mm^3^ (IQR 94–453). A total of 149 patients (49%) had a history of PI use prior to DRV, and 157 (51%) were PI-naïve. The most common previous PIs were lopinavir, atazanavir, and saquinavir. Baseline DRV RAMs were present in 1 (0.6%) PI-naïve and 20 (13.4%) PI-experienced patients. Mutations emerged under DRV pressure in a further 3 (1.9%) PI-naïve patients, and in 7 (4.7%) PI-experienced patients, 5 of whom had other DRV RAMs present at baseline (Table 1). The median time from the start of DRV to the repeat test was 196 days for PI-naïve patients and 296 days for PI-experienced.

**Conclusions:**

PI-experienced patients had a greater prevalence of DRV RAMs at baseline than PI-naïve individuals, probably due to the fact that some DRV RAMs can be selected by other PIs. This group also accumulated more RAMs during DRV exposure, possibly because previous PIs had caused minority variants which then emerged on DRV therapy. Overall, only 10 patients accumulated 16 RAMs, which supports the perception that DRV has a high genetic barrier to resistance. Repeat genotyping in the case of virological failure on DRV may still be warranted to detect emerging resistance and guide management decisions.

**Table 1:** Baseline and emerging IAS-USA DRV mutations

	PI-naive (n=157 patients)		PI-experienced (n=149 patients)	
	
IAS-USA DRV mutation	Baseline (n)	Emerged (n)	Baseline (n)	Emerged (n)
11I	0	0	1	2
32I	0	1	1	3
33F	0	0	11	2
47V	0	0	2	0
50V	0	0	1	0
54L	0	0	2	2
54M	0	0	0	0
74P	1	0	1	0
76V	0	1	5	2
84V	0	1	12	1
89V	0	0	4	1
Total mutations	1	3	40	13
Total patients	1	3	20	7
